# Decisive role of histopathology of lens capsule in the diagnosis of chronic fungal postoperative endophthalmitis

**DOI:** 10.1186/s40942-022-00370-8

**Published:** 2022-03-07

**Authors:** Mathias Paulo Loredo e Silva, Rodrigo Magno da Silva Oliveira, Bruno Freitas Lage, Clóvis Arcoverde Freitas, Moisés Salgado Pedrosa, Márcio Bittar Nehemy, Daniel Vítor Vasconcelos-Santos

**Affiliations:** 1 Hospital São Geraldo/Hospital das Clínicas da Universidade Federal de Minas Gerais, Av. Alfredo Balena 190. Sala 199, Belo Horizonte, MG 30130-100 Brazil; 2 Departamento de Oftalmologia e Otorrinolaringologia – Faculdade de Medicina da Universidade Federal de Minas Gerais, Belo Horizonte, Brazil; 3 Departamento de Anatomia Patológica e Medicina Legal – Faculdade de Medicina da Universidade Federal de Minas Gerais, Belo Horizonte, Brazil

**Keywords:** Chronic endophthalmitis, Fungi, Cataract surgery, Intraocular lens, Intraocular inflammation, Uveitis

## Abstract

We report three cases of refractory chronic endophthalmitis after cataract surgery presenting to a referral center, and with repeated negative cultures. Initial treatment consisted of intravitreal and systemic antibiotics, with partial improvement. After subsequent worsening, *pars plana* vitrectomy, intraocular lens explantation and *en bloc* capsulectomy were performed. Histopathological examination revealed multiple filamentous fungal structures, sequestered between anterior/posterior lens capsule in all cases. Chronic postoperative fungal endophthalmitis may manifest with negative cultures possibly associated with sequestration of the microorganism into the capsular bag. Careful histopathological examination of lens capsule in these cases may be essential for a definite diagnosis.

## Background

Infectious endophthalmitis is characterized by intraocular inflammation in response to invasion of ocular tissue by bacteria, fungi, parasites, protozoa or viruses. Most cases are exogenous, with microorganisms being introduced into the eye after trauma, surgery or ocular surface [1]. Frequently, postoperative endophthalmitis is acute, presenting within hours or days from the insult. Conversely, chronic postoperative endophthalmitis manifests as an indolent persistent inflammation, usually with late onset [1, 4, 8]. Fungal agents may be implicated in 20% of these cases [4].

Etiological diagnosis of endophthalmitis is based on microscopic examination and culture of vitreous and aqueous humor. Recently, polymerase chain reaction-based assays have been employed to detect microorganism DNA in these samples. In some cases, pathologic examination of the vitreous and even of lens capsule may help confirm the diagnosis [4].

This paper aims to report three cases of chronic refractory postoperative endophthalmitis with repeated negative cultures of vitreous samples, for which histopathological examination of the lens capsule defined fungal etiology.

## Report of cases

### Clinical presentation

**Fig. 1:**
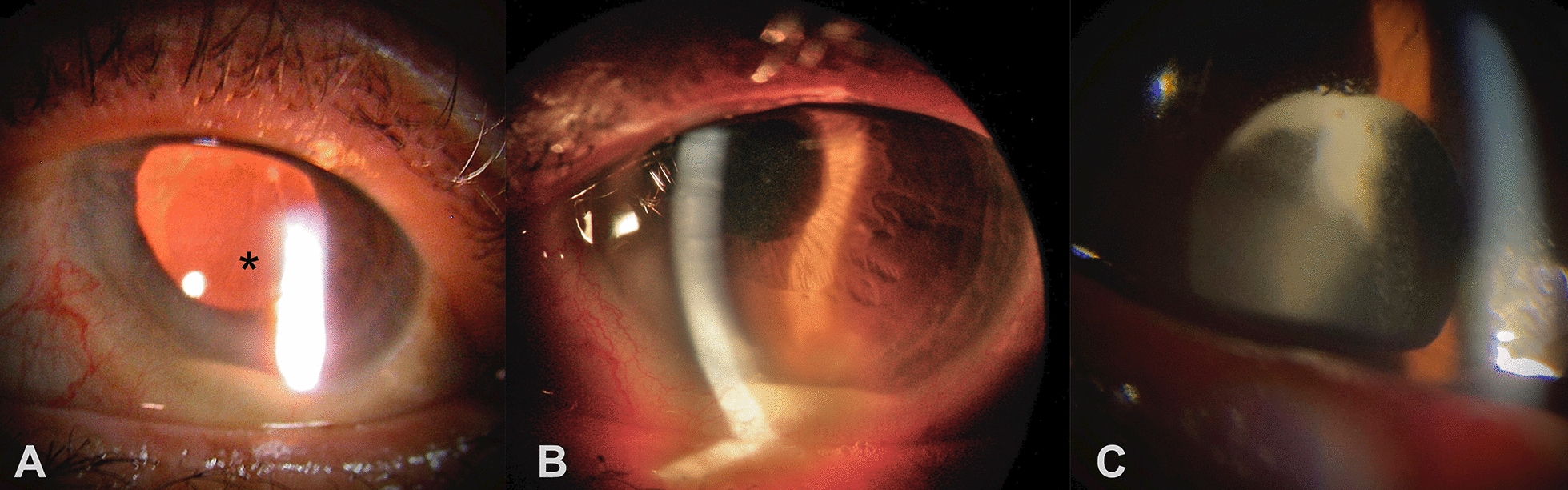
Aspect of the anterior segment of the three patients. **A** *Case 1*: a hypopyon is seen in the left eye, with retroillumination delineating an inflammatory plaque at the inferotemporal aspect of the posterior lens capsule (asterisk). **B** *Case 2*: 4 + inflammatory cells in the anterior chamber and a hypopyon are seen. **C** *Case 3*: Infiltration is observed in superior aspect of the anterior capsule, with exudation to the anterior vitreous

CASE 1. A 66-year-old female had onset of decreased vision, photophobia and redness, two months after uneventful phacoemulsification with intraocular lens (IOL) implantation in the left eye (OS). At presentation, best-corrected visual acuity (BCVA) in OS was hand motion. Slit lamp examination (SLE) showed mild diffuse conjunctival hyperemia, clear cornea with fine precipitates, 2 + flare and 4 + cells in the anterior chamber (AC). A 1.5-mm-hypopyon could also be seen, in association to a whitish plaque in the posterior lens capsule (Fig. 1A). The visualization of the posterior follow-up was unfeasible, and B-scan revealed multiple confluent vitreal membranes more concentrated inferiorly, and consistent with endophthalmitis. SLE of fellow eye was unremarkable and intraocular pressure was within normal limits in both eyes. Pars plana vitrectomy, intraocular lens explantation and en bloc capsulectomy were performed. No further surgeries were necessary, with last recorded best-corrected visual acuity of 20/40.

CASE 2. A 67-year-old male had ocular pain and decreased vision 40 days after uneventful phacoemulsification with IOL implantation in the right eye (OD). BCVA was counting fingers in OD and SLE revealed mild conjunctival hyperemia and a relatively clear cornea, with Descemet's membrane folds. AC displayed 2 + flare and 4 + cells, with formation of a 1-mm-hypopyon (Fig. 1B). After pupillary dilation, whitish intracapsular exudate could also be seen. The visualization of the posterior follow-up was unfeasible. Examination of the fellow eye was unremarkable. Intraocular pressure was normal in both eyes. Pars plana vitrectomy, intraocular lens explantation and en bloc capsulectomy were performed, and later followed by secondary intraocular lens implantation leading to final best-corrected visual acuity of 20/100.

CASE 3. An 88-year-old male presented with history of ocular pain and blurred vision 3 months after phacoemulsification with intraocular lens implantation in OD elsewhere. He had been seen 1 month before, with significant intraocular inflammation, receiving topical steroids, with insufficient response. BCVA was 20/320 in OD. SLE showed conjunctival hyperemia, 2 + cells and 1 + flare in AC and 3 + cells in anterior vitreous. A whitish plaque could also be seen behind the IOL superiorly, with a fluffy extension towards the visual axis (Fig. 1C). Intraocular pressure was normal in both eyes. Fundus examination showed atrophic unspecific chorioretinal scars and intraocular lens was within normal limits. Examination of the fellow eye was unremarkable. Pars plana vitrectomy was performed with explantation of the intraocular lens and capsular bag. No further surgeries were necessary, with final visual acuity of 20/1600.

### Procedures and histopathological examination of excised lens capsule

Initial treatment for the three patients consisted of intravitreal and systemic antibiotics (vancomycin 1 mg, ceftazidime 2.25 mg, supplemented with dexamethasone 0.4 mg), with partial improvement. After subsequent worsening and successive negative cultures of vitreous samples, *pars plana* vitrectomy, intraocular lens explantation and *en bloc* capsulectomy were performed.

**Fig. 2:**
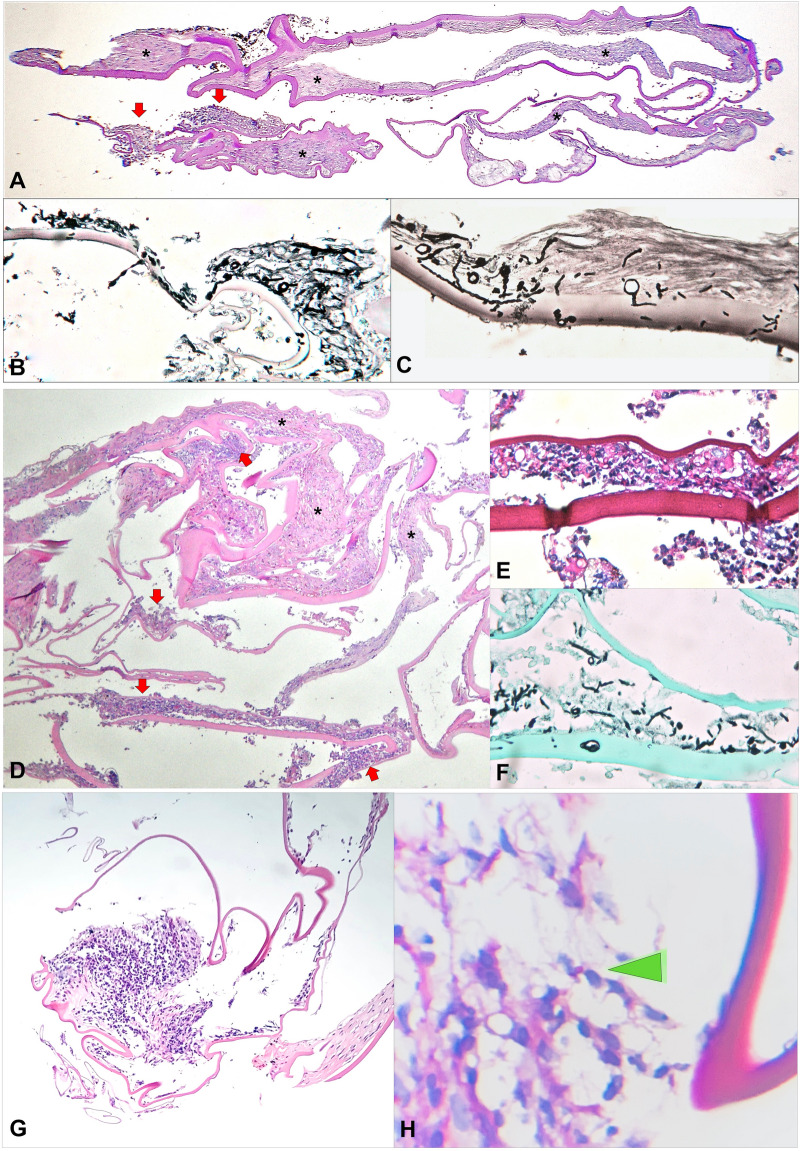
Histopathological examination of the lens capsule of the three patients. Case 1: **A** Low magnification (PAS [Periodic acid Schiff] staining) shows areas of fibrosis of anterior and posterior lens capsule (black arsterisks), as well as localized inflammatory infiltration with formation of microabscess (red arrows). **B**, **C** On Grocott methenamine silver staining, multiple fungal structures are seen sequestered between the anterior and posterior lens capsule on higher magnification. Case 2:** D** Low magnification image (hematoxylin–eosin [HE] staining) displays dense inflammatory cell infiltration (red arrows), intermingled with foci of fibrosis (black asterisks). **E**, **F** PAS **E** and Grocott **F** staining reveal numerous septate fungal hyphae within the capsular bag, sequestered between anterior and posterior lens capsule. Case 3: **G** Low magnification (HE staining) shows focus of inflammatory infiltration (red arrow) and area of fibrosis (black asterisk). **H** On high magnification, a round PAS positive structure consistent with fungal element is disclosed (green arrowhead); vitreal culture eventually grew *Acremonium sp*

Histopathological examination revealed inflammatory infiltration in the lens capsule, as well as focal areas of fibrosis (Fig. 2A, D and G). Cases 1 and 2 had multiple septate hyphae sequestered between anterior and posterior lens capsule (Fig. 2B–C and E–F). Case 3 had scarce round PAS-positive structures consistent with fungi within the lens capsule (Fig. 2H). For this case, culture eventually grew *Acremonium sp.*

Treatment with intravitreal (200 μg/0.1 mL) and oral voriconazole (200 mg bid) for several weeks eventually controlled the infectious process in all patients.

## Discussion

Chronic postoperative endophthalmitis is frequently caused by *Cultibacterium* (formerly *Propionibacterium*) *acnes*, and less often by other *Gram* positive bacteria and even fungi [1–3].

The presence of an intracapsular whitish inflammatory plaque, associated to sequestration of the microorganism, is particularly characteristic of infection by *C. acnes* [1, 4]. We report three such cases, in which this possibility was initially entertained. However, as conventional therapy failed, a more radical surgical approach was necessary. Indeed, microorganisms retained in the capsular bag are difficult to eradicate with conservative treatment, possibly resulting in recurrent/relapsing intraocular inflammation [4, 5].

Postoperative fungal endophthalmitis remains a challenge, not only because of difficulties in definition of etiologic diagnosis, but also in response to antifungal therapy. It often presents more insidiously than bacterial endophthalmitis and diagnosis can be delayed, causing high morbidity, as seen in these cases [6]. Most common fungal agents are filamentous fungi of genera *Aspergillus* and *Fusarium*. Inadvertent use of corticosteroids may also be another complicating factor [7, 8].

We initially opted for conservative therapy with systemic and sequential intravitreal antibiotics. However, cultures were repetitively negative and intraocular inflammation persisted, so that a more invasive approach was then necessary, with pars plana vitrectomy, *en bloc* capsulectomy and explantation of intraocular lens. This more aggressive procedure eventually allowed definition of fungal etiology and subsequent control of intraocular inflammation in all patients. An alternative approach would have been to perform only pars plana vitrectomy and injection of antibiotics. However, recurrence rate is significantly decreased when total capsulectomy and intraocular lens removal/exchange are added [4].

Clinicopathological correlation of the whitish capsular exudate with fungal infection of the capsular bag suggested intracapsular sequestration of fungi in these cases (Fig. 2). Surgical treatment was supplemented with intravitreal and oral voriconazole, the latter for several weeks, as previously reported for chronic postoperative fungal endophthalmitis [6].

In conclusion, chronic postoperative fungal endophthalmitis can manifest with repeated negative cultures associated with the sequestration of the microorganism in the capsular bag, similarly to what occurs in *C. acnes* endophthalmitis. Careful histopathological examination of the lens capsule in these cases may be essential for a definitive diagnosis, allowing clinicopathological correlation, and guiding appropriate treatment.
